# *Ehrlichia ruminantium*, Sudan

**DOI:** 10.3201/eid1111.050744

**Published:** 2005-11

**Authors:** Yasukazu Muramatsu, Shin-ya Ukegawa, Abdel Rahim Mohamed El Hussein, Magdi Badawi Abdel Rahman, Khalil Mohamed Ali Abdel Gabbar, Agnes Mumbi Chitambo, Tomoyoshi Komiya, Enala Tembo Mwase, Chiharu Morita, Yutaka Tamura

**Affiliations:** * Rakuno Gakuen University, Hokkaido, Japan; † Center of Veterinary Research Laboratories, Khartoum, Sudan; ‡ University of Bahr El Gazal, Khartoum, Sudan; § University of Namibia, Windhoek, Namibia; ¶ Kitasato Institute Research Center for Biologicals, Saitama, Japan; # University of Zambia, Lusaka, Zambia

**Keywords:** Ehrlichia ruminantium, Sudan, novel genotype, pCS20, *map1*, *Amblyomma* ticks, letter

**To the Editor:** *Ehrlichia ruminantium*, the causative agent of heartwater, is transmitted by *Amblyomma* spp. ticks. *Amblyomma variegatum* ticks, which are found in the Caribbean and sub-Saharan Africa, except in certain areas of southern Africa, are major vectors of *E. ruminantium* (*1**–**3*). *A. lepidum* is also an important vector of heartwater, especially in eastern Sudan (*4*). However, few epidemiologic data exist on infection rates of *Amblyomma* spp. ticks and distribution of *E. ruminantium* in Sudan. A polymerase chain reaction (PCR) assay that uses DNA probe pCS20 has been developed for detecting *E. ruminantium* (*5*). Another PCR assay for the major antigen protein 1 gene (*map1*) has been used to differentiate strains of *E. ruminantium* (*6**,**7*). These PCR assays have high sensitivity and specificity for the amplification of *E. ruminantium* DNA (*6**,**8*). For epidemiologic study of *E. ruminantium* in Sudan, we used PCR to detect *E. ruminantium* DNA in ticks. We also sequenced PCR products to identify the genotype of *E. ruminantium*.

The pCS20 DNA fragment of *E. ruminantium* was detected in 8 (8.2%) of 97 *A. variegatum* ticks and 2 (1.9%) of 106 *A. lepidum* ticks (χ^2^= 3.123, by Yates correction). The nucleotide sequences (279 bp) obtained from 5 *A. variegatum* ticks and 1 *A. lepidum* tick were identical (GenBank accession no. AB218277). The sequences were similar to those of Welgevonden, Vosloo, and Ball3 strains from southern Africa and Gardel strain from the Caribbean islands (similarity = 99.64%). The pCS20 sequences obtained in this study were different from those of strains from western Africa.

An 855-bp *map1* nucleotide sequence obtained from 1 *A. lepidum* tick was provisionally named Gedaref (GenBank accession no. AB218278). The nucleotide sequence of Gedaref was found to be closely related to those of Senegal and Pokoase strains from western Africa and to South Africa Canine and Kümm1 strains from southern Africa (similarity = 90.53%–97.43%). Gedaref clustered with these 4 strains and with 6 other strains, including Kiswani from eastern Africa and Antigua from the Caribbean islands (Figure). In contrast, the nucleotide sequence of Gedaref showed 84.8% similarity with that of Um Banein, which has been known as the only strain of *E. ruminantium* in Sudan. Um Banein formed another cluster with Gardel, Lutale, and Umpala strains from southern Africa (Figure). The *map1* coding sequence of Gedaref was closely related to those of strains Senegal, Ball3, South Africa Canine, and Pokoase (similarity = 92.61%–97.97%). Gedaref and these 4 strains formed a cluster and branch with Um Banein (similarity = 87.6%).

**Figure:**
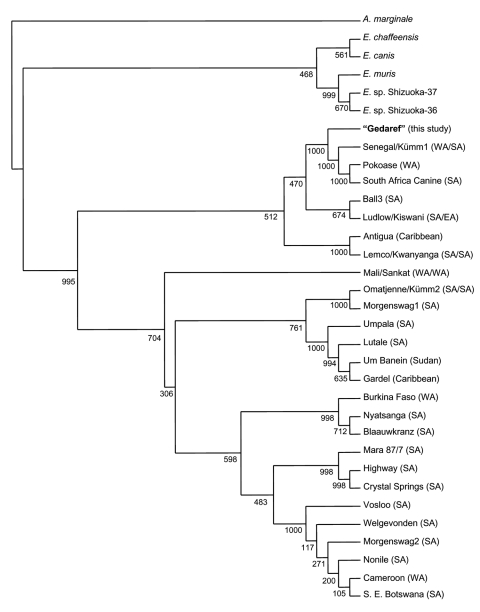
Neighbor-joining phylogram based on map1 nucleotide sequences of Ehrlichia ruminantium strains. Ninety-seven Amblyomma variegatum ticks were obtained from cattle in the suburbs of Juba in southern Sudan, and 106 A. lepidum ticks were obtained from camels in the suburbs of Gedaref in eastern Sudan in 2000. The amplicon used included all 3 variable regions in the map1 sequence (nucleotide positions 472–1377) (*7*). The nucleotide position refers to GenBank accession no. X74250. The amplicon without primer sequences (855 bp) was subjected to sequencing analysis. Sequence homogeneity was determined and multiple alignment analyses were conducted as previously described (*9*). A. marginale strain Pawhuska major surface protein 4 (GenBank accession no. AY127078) was used as an outgroup. WA, western Africa; SA, southern Africa; EA; eastern Africa. Kiswani is identical to Ludlow, Kümm1 is identical to Senegal, Kümm2 is identical to Omatjenne, Kwanyanga is identical to Lemco, and Sankat is identical to Mali (*6*).

The novel *E. ruminantium* genotype Gedaref was detected in *A. lepidum* by PCR assays. This work has shown that another strain of *E. ruminantium*, in addition to the Um Banein strain, is present in Sudan. Since the Um Banein strain of *E. ruminantium* was isolated from sheep in 1984 (*4*), no other strain had been reported in Sudan. The *map1* sequence of Gedaref has diverged from that of Um Banein. Gedaref formed a cluster with several strains that originated in southern and western Africa. As previously reported, the variation of *map1* sequences of *E. ruminantium* strains does not reflect the geographic distribution of the strains (*6*). However, Gedaref was distinctively differentiated from the Um Banein strain. Gedaref was closely related to 3 southern African strains and a Caribbean strain in the pCS20 sequence but different from western African strains. The pCS20 sequence has been highly conserved among strains from western Africa (*10*), and the distribution of *A. lepidum* is limited to eastern Africa (*3*). If one considers the distribution of *A. lepidum*, results of genetic analyses in the pCS20 gene regions of Gedaref are important for epidemiologic research on *E. ruminantium*.

We detected pCS20 DNA specific for *E. ruminantium* in *A. variegatum*. This tick is widely distributed in Africa and is the most efficient vector of heartwater (*3*). Nevertheless, detection of *E. ruminantium* in *A. variegatum* in Sudan had not previously been reported. Our results show that *A. variegatum* is also an important vector of *E. ruminantium* in Sudan. *A. variegatum* ticks are also found North and South America, Southeast Asia, and Australia (*3*). Thus, the potential spread of *E. ruminantium* to livestock is a continuous threat in these regions from the importation of tick-infested and subclinically infected wild animals from Africa and the importation of livestock from Caribbean islands (*1**,**2*). We believe that *A. variegatum* plays an important role in the spread of heartwater because of its ability to adapt to various environments.

## References

[1] Uilenberg G. Heartwater (*Cowdria ruminantium* infection): current status. Adv Vet Sci Comp Med. 1983;27:427–80. 6359836

[2] Burridge MJ, Simmons LA, Peter TF, Mahan SM. Increasing risks of introduction of heartwater onto the American mainland associated with animal movements. Ann N Y Acad Sci. 2002;969:269–74. 10.1111/j.1749-6632.2002.tb04391.x 12381604

[3] Walker JB, Olwage A. The tick vectors of *Cowdria ruminantium* (Ixodoidea, Ixodidae, genus *Amblyomma*) and their distribution. Onderstepoort J Vet Res. 1987;54:353–79. 3329325

[4] Jongejan F, Morzaria SP, Shariff OA, Abdalla HM. Isolation and transmission of *Cowdria ruminantium* (causal agent of heartwater disease) in Blue Nile Province, Sudan. Vet Res Commun. 1984;8:141–5. 10.1007/BF02214705 6740919

[5] Peter TF, Deem SL, Barbet AF, Norval RAI, Simbi BH, Kelly PJ, Development and evaluation of PCR assay for detection of low levels of *Cowdria ruminantium* infection in *Amblyomma* ticks not detected by DNA probe. J Clin Microbiol. 1995;33:166–72. 7699036 10.1128/jcm.33.1.166-172.1995 PMC227901

[6] Allsopp MTEP, Dorfling CM, Maillard JC, Bensaid A, Haydon DT, van Heerden H, *Ehrlichia ruminantium* major antigenic protein gene (*map1*) variants are not geographically constrained and show no evidence of having evolved under positive selection pressure. J Clin Microbiol. 2001;39:4200–3. 10.1128/JCM.39.11.4200-4203.2001 11682561 PMC88518

[7] Reddy GR, Sulsona CR, Harrison RH, Mahan SM, Burridge MJ, Barbet AF. Sequence heterogeneity of the major antigenic protein 1 genes from *Cowdria ruminantium* isolates from different geographical areas. Clin Diagn Lab Immunol. 1996;3:417–22. 8807206 10.1128/cdli.3.4.417-422.1996 PMC170360

[8] Peter TF, Barbet AF, Alleman AR, Simbi BH, Burridge MJ, Mahan SM. Detection of the agent of heartwater, *Cowdria ruminantium*, in *Amblyomma* ticks by PCR: validation and application of the assay to field ticks. J Clin Microbiol. 2000;38:1539–44. 10747140 10.1128/jcm.38.4.1539-1544.2000 PMC86485

[9] Morita C, Hussein ARME, Matsuda E, Gabbar KMAA, Muramatsu Y, Rahman MBA, Spotted fever group rickettsiae from ticks captured in Sudan. Jpn J Infect Dis. 2004;57:107–9. 15218219

[10] Allsopp MTEP, van Heerden H, Steyn HC, Allsopp BA. Phylogenetic relationships among *Ehrlichia ruminantium* isolates. Ann N Y Acad Sci. 2003;990:685–91. 10.1111/j.1749-6632.2003.tb07444.x 12860707

